# Tracking of Neuroinflammation Dynamics During Combined Anti-β-Amyloid Therapy (AAT) and Immunomodulation in a Preclinical Alzheimer’s Disease Model

**DOI:** 10.3390/ijms27104632

**Published:** 2026-05-21

**Authors:** Karin Wind-Mark, Lea H. Kunze, Michael Willem, Giovanna Palumbo, Camilla Giudici, Brigitte Nuscher, Guido Boening, Franz J. Gildehaus, Simon Lindner, Rudolf A. Werner, Nicolai Franzmeier, Johannes S. Gnörich, Matthias Brendel, Artem Zatcepin

**Affiliations:** 1 Department of Nuclear Medicine, LMU University Hospital, LMU Munich, 81377 Munich, Germany; karin.wind@med.uni-muenchen.de (K.W.-M.); giovanna.palumbo@med.uni-muenchen.de (G.P.); guido.boening@med.uni-muenchen.de (G.B.); franz.gildehaus@med.uni-muenchen.de (F.J.G.); simon.lindner@med.uni-muenchen.de (S.L.); rudolf.werner@med.uni-muenchen.de (R.A.W.); johannes.gnoerich@med.uni-muenchen.de (J.S.G.); artem.zatcepin@med.uni-muenchen.de (A.Z.); 2 Munich Cluster for Systems Neurology (SyNergy), 81377 Munich, Germany; nicolai.franzmeier@med.uni-muenchen.de; 3 Biomedical Center (BMC), Division of Metabolic Biochemistry, Faculty of Medicine, LMU Munich, 81377 Munich, Germany; michael.willem@mail03.med.uni-muenchen.de (M.W.); brigitte.nuscher@mail03.med.uni-muenchen.de (B.N.); 4 German Center for Neurodegenerative Diseases (DZNE) Munich, 81377 Munich, Germany; camilla.giudici@mail03.med.uni-muenchen.de; 5 Russell H. Morgan Department of Radiology and Radiological Sciences, Johns Hopkins School of Medicine, Baltimore, MD 21287, USA; 6 Institute for Stroke and Dementia Research (ISD), LMU University Hospital, LMU Munich, 81377 Munich, Germany; 7 Department of Psychiatry and Neurochemistry, Institute of Neuroscience and Physiology, The Sahlgrenska Academy, University of Gothenburg, SE 413 90 Gothenburg, Sweden; 8 German Cancer Research Center (DKFZ), 69120 Heidelberg, Germany; 9 German Cancer Consortium (DKTK), Partner Site Munich, 80336 Munich, Germany; 10 Bavarian Cancer Research Center (BZKF), 91052 Erlangen, Germany; 11 Department of Nuclear Medicine, Hannover Medical School, 30625 Hannover, Germany

**Keywords:** Alzheimer’s disease, microglia, monoclonal antibody, pioglitazone, neuroinflammation, TSPO-PET, desynchronization index

## Abstract

Neuroinflammation is increasingly recognized as a key modulator of therapeutic response and adverse events in Alzheimer’s disease (AD), especially during anti-amyloid-β (Aβ) monoclonal antibody (Aβ-mAb) treatment. We applied longitudinal translocator protein (TSPO) positron emission tomography (PET) to evaluate TSPO-associated neuroinflammatory responses to chronic Aβ-mAb therapy and their modulation by the peroxisome proliferator-activated receptor γ (PPARγ) agonist pioglitazone. App*^NL-G-F^* knock-in mice underwent TSPO-PET and Aβ-PET imaging at 5, 7.5, and 10 months of age across four treatment arms: placebo, Aβ-mAb, pioglitazone, and combination therapy. TSPO-PET detected early and progressive neuroinflammatory responses to Aβ-mAb that appeared lower with pioglitazone co-treatment. Both mono- and combination therapy were associated with altered temporal and spatial dynamics of the TSPO-PET signal. In addition, we applied a previously validated microglia desynchronization index based on TSPO-PET connectivity, which captured individual variation in regional TSPO-PET organization and correlated with cognitive performance. Together, TSPO-PET and its regional synchronicity can quantify longitudinal, region-specific treatment effects, which may help differentiate harmful from adaptive neuroinflammatory responses. These findings highlight the potential of TSPO-PET as a stratification biomarker to optimize therapeutic interventions. TSPO-PET therefore enables in vivo tracking of treatment-associated neuroinflammatory responses during anti-Aβ immunotherapy and provides a non-invasive framework for evaluating combination strategies targeting amyloid pathology and immune regulation in AD.

## 1. Introduction

Alzheimer’s disease (AD) affects around 416 million people worldwide, or 22% of all people older than 50 years, when preclinical and prodromal stages are included, with the majority in the asymptomatic preclinical stage [1]. Anti-amyloid-β (Aβ) monoclonal antibodies (mAbs) such as aducanumab, lecanemab, and donanemab initiated a new therapeutic era in AD. Aβ-mAbs produce robust plaque clearance as shown by β-amyloid positron emission tomography (Aβ-PET) and result in approximately 30% slowing of cognitive decline [2]; however, they are associated with amyloid-related imaging abnormalities (ARIA)—MRI-visible changes reflecting brain swelling or fluid accumulation (ARIA-E) and small areas of bleeding or iron deposition (ARIA-H)—which are often mild or asymptomatic but can occasionally cause headache, confusion, or focal symptoms and therefore require routine MRI monitoring and, when detected, dose interruption or adjustment [3,4].

Neuroinflammation is now recognized as a crucial modulator of AD pathogenesis [5,6]. Activated microglia can facilitate Aβ-mAb-mediated plaque clearance, but excessive Fc-receptor signaling and complement activation may amplify vascular permeability and precipitate ARIA-E/H [7,8,9], highlighting a narrow therapeutic window between beneficial and harmful neuroinflammation. The 18 kDa translocator protein (TSPO) is an outer mitochondrial membrane protein that is strongly upregulated in activated microglia in mice, although the TSPO-PET signal may also include astrocytic or vascular contributions [10]; TSPO can be monitored using PET. Accordingly, throughout the study, we interpret TSPO-PET primarily as a TSPO-associated neuroinflammatory signal, while avoiding cell-specific conclusions in the absence of histological co-localization. Unlike in humans, rodent TSPO lacks the rs6971 affinity polymorphism, simplifying quantification [11]. Longitudinal TSPO-PET in AD models tracks plaque-associated microgliosis and correlates with histology and cytokines [12], supporting its use for disease staging and pharmacodynamic monitoring [13].

Pioglitazone, a peroxisome-proliferator-activated-receptor-γ (PPARγ) agonist approved for type 2 diabetes, switches microglia toward an anti-inflammatory, Aβ-resolving phenotype [14,15]. In App*^NL-G-F^* and PS2APP mice, chronic pioglitazone treatment shifted plaques to a more fibrillar state while improving synaptic density and spatial learning [16]. Notably, the individual response to chronic pioglitazone-induced immunomodulation is influenced by baseline microglial activation and sex, as shown by our longitudinal TSPO PET study [17].

In this work, we combined an Aβ-mAb with continuous pioglitazone co-therapy in the App*^NL-G-F^* knock-in mouse model and paired [^18^F]GE-180, also known as [^18^F]flutriciclamide, and TSPO-PET with [^18^F]florbetaben ([^18^F]FBB) Aβ-PET. This dual-tracer design allowed us to assess treatment-induced changes in the TSPO-associated neuroinflammatory signal together with changes in Aβ-PET tracer binding. We questioned whether the immunomodulatory profile of pioglitazone could preserve the plaque-clearing efficacy of the Aβ-mAb while mitigating the related TSPO-PET response. We aimed to provide a preclinical rationale for adjunctive PPARγ stimulation as a strategy to widen the therapeutic window of anti-Aβ immunotherapy.

In our previous work [18], we provided the first evidence that microglial activation is regionally synchronized in the healthy brain but undergoes regional desynchronization with ongoing neurodegenerative disease, and we developed a personalized microglia desynchronization index (DI) that quantifies the extent to which a subject deviates from the microglia synchronicity pattern of a control cohort. The biological basis of this TSPO-PET-derived metric was supported by pharmacological microglia depletion, models of dysfunctional microglia, AD mouse models, human AD-continuum data, and single-cell radiotracing, which identified microglia as the dominant cellular contributor to regionally desynchronized TSPO tracer uptake in App*^NL-G-F^* mice. We therefore used DI in this work as a complementary, previously validated PET-based biomarker and correlated it with mouse cognitive performance.

## 2. Results

### 2.1. TSPO-PET Signals Are Increased in App^NL-G-F^ Mice Compared to Wild-Type Mice and Indicate Exploratory Treatment-Related Differences in the App^NL-G-F^ Mouse Model

**Figure 1:**
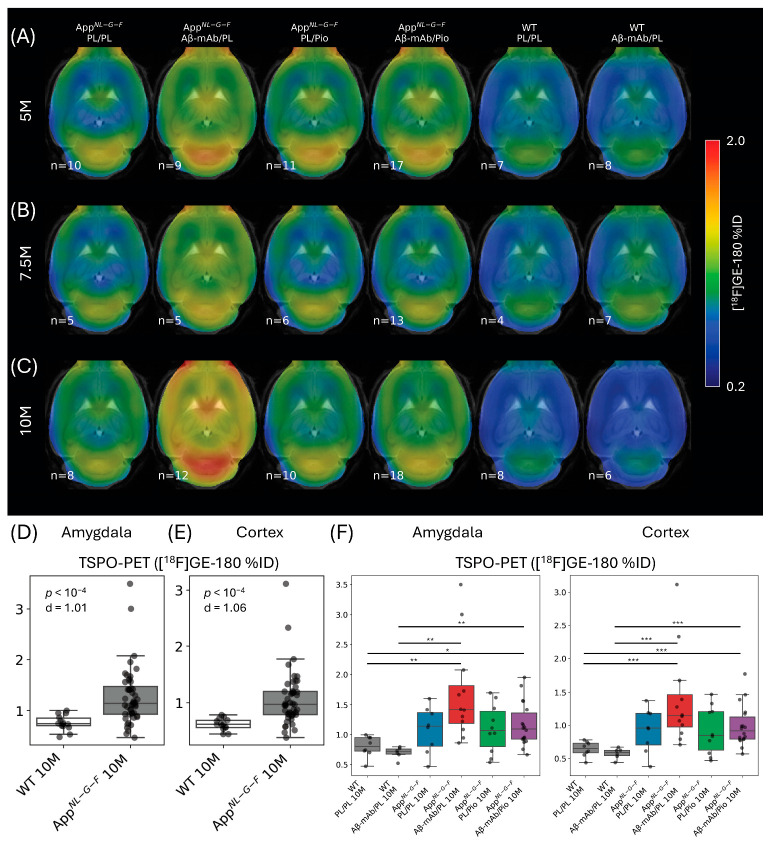
(**A**–**C**). Voxelwise group average translocator protein positron emission tomography (TSPO-PET) images (percentage of injected dose per cubic centimeter [%ID] normalization) generated from all animals within each cohort at (**A**) 5 months, (**B**) 7.5 months, and (**C**) 10 months of age. Treatment arms: PL/PL—double placebo, Aβ-mAb/PL—anti-β-amyloid antibodies, PL/Pio—pioglitazone, Aβ-mAb/Pio—combination of anti-β-amyloid antibodies and pioglitazone. WT—wild-type mice, App*^NL-G-F^*—APP knock-in mice. The number of mice in respective cohorts (*n*) is shown in the bottom left corner. (**D**,**E**) Global TSPO-PET (%ID) at 10 months of age, pooled across treatment arms within each genotype (WT, *n* = 14; App*^NL-G-F^*, *n* = 48) to illustrate the overall genotype effect in the (**D**) amygdala and (**E**) cortex. (**F**) Treatment-arm-specific comparisons indicate the highest TSPO-PET signals for anti-Aβ-mAb monotherapy in App*^NL-G-F^* mice, without reaching significance for the contrast against other App*^NL-G-F^* groups after false-discovery-rate correction. Significance annotations (Mann-Whitney U test, post-hoc false-discovery rate (FDR)-adjusted *p*-values): * *p* < 0.05, ** *p* < 0.01, *** *p* < 0.001. Boxes show the interquartile range (IQR) and median; whiskers follow the 1.5 × IQR rule.

To investigate the effects of the different treatment arms on neuroinflammation and Aβ plaques in the brain, we analyzed TSPO-PET and Aβ-PET data from App*^NL-G-F^* and wild-type (WT) mice. This analysis aimed to target brain-wide effects. TSPO-PET was quantified as the percentage of injected dose per cubic centimeter (%ID/cc, further referred to as %ID) to avoid potential bias from pseudo-reference-region assumptions, whereas Aβ-PET was quantified as the standardized uptake value ratio (SUVR) using the established periaqueductal gray matter pseudo-reference region for [^18^F]FBB (as specified in Section 4.6). Figure 1 and Figure S1 present horizontal slices of the voxelwise group average [^18^F]GE-180 and [^18^F]FBB images, respectively, generated from all animals within each study cohort at 5, 7.5, and 10 months of age. As expected from previous studies [16], Aβ-PET signals increased in the pioglitazone treatment group, consistent with treatment-associated changes in plaque properties or tracer binding (Figure S1), which was also observed for the combination treatment with the anti-Aβ-mAb. As shown in Figure 1A–C, TSPO-PET signals were higher in App*^NL-G-F^* mice compared to WT mice at all timepoints. By 10 months of age, the TSPO-PET signal was significantly higher in the entire cohort of App*^NL-G-F^* mice compared to WT mice (Figure 1D, amygdala, *p* < 10^−4^, d = 1.0 (+72.5%); Figure 1E, cortex, *p* < 10^−4^, d = 1.06 (+67.3%)). The differences between the genotypes were also confirmed by Kruskal-Wallis H-test: amygdala H(5) = 21.3, *p* = 0.0007; cortex H(5) = 23.6, *p* = 0.0003 (Figure 1F).

Treatment-arm-specific global contrasts are shown in Figure 1F and are interpreted descriptively, as these comparisons did not remain significant after false-discovery rate (FDR) correction. Among the App*^NL-G-F^* cohorts, mice that received monotherapy with the anti-Aβ-mAb displayed the highest TSPO-PET signal at all ages, indicative of an elevated TSPO-associated neuroinflammatory signal, which was most pronounced at 10 months (Figure 1F, amygdala: +56% compared to placebo, *p* = 0.08, d = 0.89; Figure 1F, cortex: +49% compared to placebo, *p* = 0.10, d = 0.78). In contrast, mice that received pioglitazone in addition to anti-Aβ-mAb did not show a notable difference in TSPO-PET signals compared to App*^NL-G-F^* controls that received placebo (amygdala: +7.2%, *p* = 0.71, d = 0.21; cortex: +7.1%, *p* = 0.71, d = 0.21). This descriptive pattern is compatible with attenuation of the treatment-associated TSPO-PET increase after anti-Aβ-mAb treatment. Similarly, mice treated with pioglitazone alone did not exhibit an increase in TSPO-PET signals compared to placebo (amygdala: −1.7%, *p* = 0.95, d = −0.05; cortex: −0.1%, *p* = 1.00, d = 0.00). Descriptively, the TSPO-PET signal was lower in the dual-treatment group compared to the anti-Aβ-mAb monotherapy group, both in the amygdala (Figure 1F, −31%, *p* = 0.09, d = −0.90) and in the cortex (Figure 1F, −28%, *p* = 0.12, d = −0.78). However, these contrasts did not reach statistical significance after FDR correction and are therefore not interpreted as confirmatory treatment effects.

In summary, we observed a higher global TSPO-PET signal in the brains of mice in the anti-Aβ-mAb monotherapy arm at 10 months of age compared to placebo and pioglitazone monotherapy, both visually (Figure 1C) and quantitatively (based on Cohen’s d). This pattern is compatible with a possible attenuation of the Aβ-mAb-associated global TSPO-PET increase by pioglitazone; however, the relevant treatment-arm contrasts did not reach significance after FDR correction, which can be explained by substantial interindividual heterogeneity in global TSPO-PET signal. Thus, these contrasts should be considered descriptive rather than confirmatory.

### 2.2. Anti-Aβ-mAb and Pioglitazone Treatments Are Associated with Reduced Longitudinal Progression of Neuroinflammation in the App^NL-G-F^ Mouse Model

**Figure 2:**
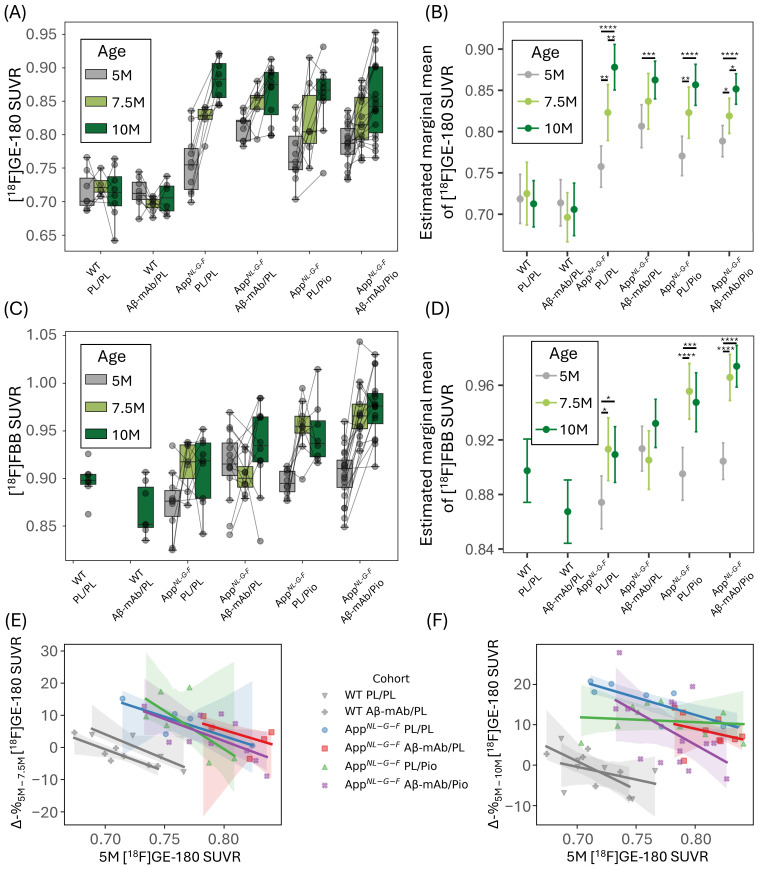
(**A**) Cortical TSPO-PET standardized uptake value ratio (SUVR) values (brain stem-scaled) across cohorts and timepoints. (**B**) Linear mixed-effects model (LMEM)-derived estimated marginal means of cortical TSPO-PET SUVR. (**C**) Cortical Aβ-PET SUVR values (periaqueductal gray matter-scaled) across cohorts and timepoints. (**D**) LMEM-derived estimated marginal means of cortical Aβ-PET SUVR. (**E**,**F**) Relationship between baseline cortical TSPO-PET SUVR (5 months) and subsequent percentage change in cortical TSPO-PET SUVR from 5 to 7.5 months (**E**) and from 5 to 10 months (**F**); lines represent linear regression fits, translucent bands indicate their 95% confidence intervals. Boxes show the IQR and median; whiskers follow the 1.5 × IQR rule. Significance annotations (post-hoc FDR-adjusted *p*-values) are shown only for within-cohort comparisons; * *p* < 0.05, ** *p* < 0.01, *** *p* < 0.001, **** *p* < 0.0001. Error bars show 95% confidence intervals. Sample sizes (*n*) per cohort at 5, 7.5, and 10 months were as follows: TSPO-PET (**A**,**B**): WT PL/PL, 7/4/8; WT Aβ-mAb/PL, 8/7/6; App*^NL-G-F^* PL/PL, 10/5/8; App*^NL-G-F^* Aβ-mAb/PL, 9/5/12; App*^NL-G-F^* PL/Pio, 11/6/10; App*^NL-G-F^* Aβ-mAb/Pio, 17/13/18. Aβ-PET (**C**,**D**): WT PL/PL, 0/0/7; WT Aβ-mAb/PL, 0/0/7; App*^NL-G-F^* PL/PL, 10/7/9; App*^NL-G-F^* Aβ-mAb/PL, 14/8/12; App*^NL-G-F^* PL/Pio, 10/9/8; App*^NL-G-F^* Aβ-mAb/Pio, 21/13/16. Panels (**E**,**F**) include only mice with data available at both relevant timepoints.

Whereas the global analysis in Section 2.1 used %ID for descriptive whole-brain comparisons of the TSPO-PET signal, the following analyses use SUVR to reduce interindividual variance and better capture regional and longitudinal treatment effects. SUVR values were calculated by normalizing the TSPO-PET images to the individual uptake in the brain stem, identified via a data-driven approach as the most suitable pseudo-reference region: it showed the smallest genotype-related difference in %ID compared to all other brain regions (Table S1, Figure S2). The resulting SUVR data were then analyzed longitudinally (Figure 2A). Moreover, we investigated a pre-established index of microglia desynchronization (DI) [18], calculated based on the SUVR values as an alternative quantification approach. Both SUVR (Figure S3A) and DI first principal component (PC1) (Figure S3B) values correlated with the original %ID-normalized values.

We assessed the effects of genotype, treatment, and age on the cortical [^18^F]GE-180 and [^18^F]FBB uptake via a linear mixed-effects model (LMEM) with random intercepts for individual mice. For [^18^F]GE-180, a type III analysis of variance (ANOVA) with Satterthwaite’s method (Table S2) revealed significant main effects of genotype (F^1, 59.81^ = 115.64, *p* < 10^−14^) and timepoint (F^2, 89.26^ = 19.00, *p* < 10^−6^), as well as a genotype × timepoint interaction (F^2, 90.59^ = 16.79, *p* < 10^−6^). For [^18^F]FBB, we observed significant main effects of genotype (F^1, 132.51^ = 12.67, *p* = 0.0005), treatment (F^3, 68.58^ = 11.66, *p* < 10^−5^), and timepoint (F^2, 91.48^ = 32.32, *p* < 10^−10^), as well as a genotype × treatment (F^1, 132.51^ = 6.04, *p* = 0.015) and treatment × timepoint (F^6, 91.47^ = 4.42, *p* = 0.0006) interaction.

Post-hoc contrasts run on estimated marginal means (Figure 2B, Table S3) of each cohort-timepoint combination demonstrated stable cortical TSPO-PET SUVR over time in both WT mice treated with placebo (5.0–7.5 M: +0.006 (+0.9%), SE = 0.021, *p* = 0.76; 5.0–10 M: −0.006 (−0.8%), SE = 0.017, *p* = 0.76) and WT mice treated with anti-Aβ-mAb (5.0–7.5 M: −0.018 (−2.5%), SE = 0.017, *p* = 0.66; 5.0–10 M: −0.008, SE = 0.018, *p* = 0.66), consistent with the lack of a significant time effect in this genotype regardless of treatment.

Among the App*^NL-G-F^* cohorts, placebo-treated mice showed the largest cortical TSPO-PET SUVR increase over time (5.0–7.5 M: +0.065 (+8.6%), SE = 0.019, *p* = 0.0011; 5.0–10 M: +0.120 (+15.9%), SE = 0.016, *p* < 0.0001), which highlights the previously reported strong time-dependent neuroinflammation of App*^NL-G-F^* mice in the absence of treatment [17]. By contrast, anti-Aβ-mAb monotherapy in App*^NL-G-F^* mice demonstrated a smaller increase in cortical TSPO-PET SUVR by 10 M (+0.056 (+6.9%), SE = 0.015, *p* = 0.0008), but no significant change in TSPO-PET signals at 7.5 M (+0.030 (+3.7%), SE = 0.019, *p* = 0.16). Pioglitazone monotherapy in App*^NL-G-F^* mice resulted in a cortical TSPO-PET signal increase at both 7.5 M (+0.052 (+6.8%), SE = 0.017, *p* = 0.0045) and 10 M (+0.086 (+11.2%), SE = 0.014, *p* < 0.0001) timepoints. Similarly, dual treatment with anti-Aβ-mAb and pioglitazone in App*^NL-G-F^* mice showed only moderate progression of neuroinflammation over time: +0.030 (+3.9%) by 7.5 M (SE = 0.013, *p* = 0.017) and +0.063 (+8.0%) by 10 M (SE = 0.011, *p* < 0.0001). This suggests that all investigated treatment options (Aβ-mAb/PL, PL/Pio, Aβ-mAb/Pio) reduce the magnitude or delay the timing of the genotype-driven increase in cortical TSPO-PET SUVR compared to placebo. In line with this interpretation, biochemical validation analysis at the terminal time point showed treatment-associated changes in Aβ- and neuroinflammation-related markers (Figure S4). Namely, using an enzyme-linked immunosorbent assay (ELISA) in brain homogenate fractions, we observed a significant reduction of insoluble Aβ40 (*p* < 0.01), insoluble Aβ42 (*p* < 0.0001), and diethylamine buffer (DEA)-soluble Aβ42 (*p* < 0.001) in the double-treatment arm as well as insoluble Aβ42 (*p* < 0.001) and DEA-soluble Aβ42 (*p* < 0.01) in the anti-Aβ-mAb monotherapy arm compared to double placebo. Triggering receptor expressed on myeloid cells 2 (Trem2) (both radio-immuno-precipitation-assay-buffer-[RIPA]- and DEA-soluble) concentration in brain homogenate was reduced across all treatment arms compared to double placebo (RIPA: Aβ-mAb/PL *p* < 0.001, PL/Pio *p* < 0.01, Aβ-mAb/Pio *p* < 0.0001; DEA: Aβ-mAb/PL *p* < 0.0001, PL/Pio *p* < 0.05, Aβ-mAb/Pio *p* < 0.0001). Additionally, ELISA-based quantification showed that insoluble apolipoprotein E (ApoE) concentration was significantly reduced in the double-treatment arm compared to double placebo (*p* < 0.001).

Placebo-treated App*^NL-G-F^* mice demonstrated only a slight increase in cortical [^18^F]FBB SUVR by 7.5 M (+0.039 (+4.5%), SE = 0.014, *p* = 0.013). App*^NL-G-F^* mice treated with anti-Aβ-mAb showed no significant increase in this parameter (+0.019 (+2.0%) by 10 M, SE = 0.011, *p* = 0.15) (Figure 2C,D; Tables S4 and S5). By contrast, pioglitazone monotherapy and dual treatment resulted in a more substantial increase in cortical [^18^F]FBB SUVR by 7.5 M (PL/Pio: +0.061 (+6.8%), SE = 0.013, *p* < 0.0001; Aβ-mAb/Pio: +0.061 (+6.7%), SE = 0.010, *p* < 0.0001), which is consistent with treatment-associated changes in Aβ-PET tracer binding, potentially reflecting altered Aβ plaque properties such as greater fibrillarity or compactness, as reported in [16].

In most cohorts, lower cortical TSPO-PET SUVR at 5 months of age was associated with a larger percentage change in cortical TSPO-PET SUVR from 5.0 to 7.5 months (Figure 2E) and from 5.0 to 10 months (Figure 2F). This negative correlation was, however, reduced by all the treatment options (5–10 M: R^2^ = 0.69 in App*^NL-G-F^* PL/PL versus R^2^ = 0.11, R^2^ = 0.02, and R^2^ = 0.27 in Aβ-mAb/PL-, PL/Pio-, and Aβ-mAb/Pio-treated App*^NL-G-F^* mice, respectively). This pattern indicates that in placebo-treated App*^NL-G-F^* mice, baseline cortical TSPO-PET SUVR strongly predicts the subsequent increase in the TSPO-associated neuroinflammatory signal, whereas in all treated groups, this dependence is markedly attenuated. This weakening of the baseline-change coupling is consistent with a treatment-related modulation of neuroinflammatory progression, although we cannot determine from this analysis alone whether the effect is preferentially expressed in mice with low baseline TSPO levels or reflects a more global reduction in variability. In other words, in untreated App*^NL-G-F^* mice, baseline TSPO-PET SUVR accounts for much of the variance in future TSPO-PET increases, which suggests that early TSPO-PET may serve as an imaging marker of susceptibility to larger subsequent TSPO-associated neuroinflammatory responses. In treated mice, this predictive relationship is largely diminished.

### 2.3. Pioglitazone Treatment Is Associated with Early Coupling Between TSPO- and Aβ-PET Signals in the App^NL-G-F^ Mouse Model

**Figure 3:**
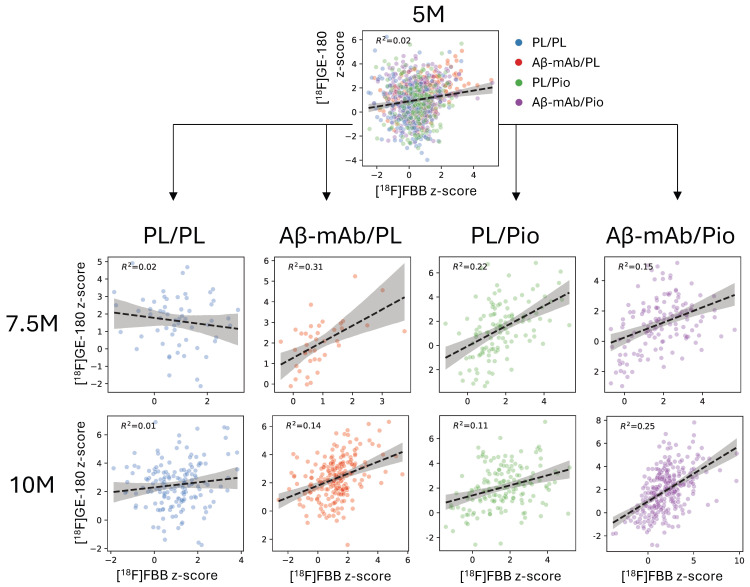
Correlations between regional mean [^18^F]florbetaben ([^18^F]FBB) and [^18^F]GE-180 z-scores in App*^NL-G-F^* mice at 5, 7.5, and 10 months of age across 20 investigated brain regions. Each point represents one brain region in one mouse. Dashed lines represent linear regression fits; shaded bands indicate their 95% confidence intervals. Sample sizes (*n* = mice) per treatment arm at 5, 7.5, and 10 months were as follows: PL/PL, 10/3/8; Aβ-mAb/PL, 9/2/11; PL/Pio, 10/6/8; Aβ-mAb/Pio, 17/7/15.

To investigate the association between the Aβ-PET signal and TSPO-associated neuroinflammatory signal during treatment, we evaluated the relationship between Aβ- and TSPO-PET in App*^NL-G-F^* mice using z-score images (relative to WT levels). Figure 3 shows correlations between the two tracers, with each point representing the mean z-score value in one of the 20 investigated brain regions in an individual mouse (see the list of regions in Table S6). At treatment start (5.0 M), only a small correlation was observed between Aβ plaque load and neuroinflammation (R^2^ = 0.02, *p* < 0.0001). At 7.5 M, all treatment options except the placebo arm led to a notable association between TSPO- and Aβ-PET signals (PL/PL: R^2^ = 0.02, *p* = 0.28; Aβ-mAb/PL: R^2^ = 0.31, *p* = 0.0002; PL/Pio: R^2^ = 0.22, *p* < 0.0001; Aβ-mAb/Pio: R^2^ = 0.15, *p* < 0.0001). At 10 M, both monotherapy arms with anti-Aβ-mAb or pioglitazone still showed a moderate regional association between both biomarkers (R^2^ = 0.14, *p* < 0.0001; R^2^ = 0.11, *p* < 0.0001, respectively), while the dual-treatment arm demonstrated the strongest association between microglial activation and fibrillar amyloidosis (R^2^ = 0.25, *p* < 0.0001). No association between microglial activation and fibrillar amyloidosis was observed in the placebo group (R^2^ = 0.01, *p* = 0.13). Thus, both anti-Aβ-mAb and pioglitazone treatments were associated with a stronger regional coupling between TSPO- and Aβ-PET signals than placebo, with the dual-treatment arm indicating the most sustained association at 10 months. Since each mouse contributed multiple regional values, these analyses were interpreted as descriptive regional coupling analyses rather than independent mouse-level treatment contrasts.

### 2.4. TSPO-PET Correlates with Behavioral Outcomes in App^NL-G-F^ Mice Treated with Aβ-mAb

**Figure 4:**
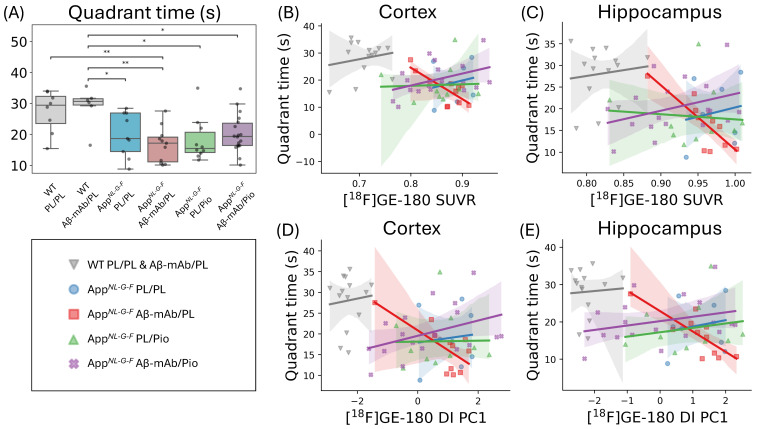
(**A**) Morris water maze performance (time spent in the target quadrant) at 10 months of age. (**B**–**E**) Associations between TSPO-PET-derived measures and behavioral performance at 10 months in the investigated cohorts: (**B**) cortical TSPO-PET SUVR, (**C**) entorhinal-hippocampus-amygdala region (EHA) TSPO-PET SUVR, (**D**) cortical desynchronization index first principal component (DI PC1), and (**E**) EHA DI PC1. Boxes show the IQR and median; whiskers follow the 1.5 × IQR rule. Lines represent linear regression fits; translucent bands indicate their 95% confidence intervals. Statistical significance: * *p* < 0.05, ** *p* < 0.01. Sample sizes (*n*) per cohort were as follows: WT PL/PL, 8; WT Aβ-mAb/PL, 6; App*^NL-G-F^* PL/PL, 8; App*^NL-G-F^* Aβ-mAb/PL, 12; App*^NL-G-F^* PL/Pio, 10; App*^NL-G-F^* Aβ-mAb/Pio, 18.

Finally, we questioned whether the investigated treatment options influenced the cognitive performance of the mouse cohorts in relation to PET biomarkers. For this purpose, we performed behavioral testing at 10 months of age using a Morris water maze (MWM). App*^NL-G-F^* mice spent significantly less time in the target quadrant than WT mice (Figure 4A). Moreover, in App*^NL-G-F^* mice that received anti-Aβ-mAb monotherapy, there was a negative correlation between quadrant time and both TSPO-PET SUVR (Figure 4B, cortex, R^2^ = 0.58; Figure 4C, entorhinal-hippocampus-amygdala region [EHA], R^2^ = 0.65) and TSPO-PET (Figure 4D, cortex, R^2^ = 0.51; Figure 4E, EHA, R^2^ = 0.65) desynchronization. No notable correlation between TSPO-PET indices and behavior was observed in App*^NL-G-F^* mice treated with placebo, pioglitazone monotherapy, or dual therapy, indicating that the negative association between TSPO-PET indices and behavioral performance was observed only in the anti-Aβ monotherapy group. This pattern is consistent with, but does not establish, a modifying effect of pioglitazone, as between-group differences in variance or dynamic range may contribute as well.

## 3. Discussion

**Figure 5:**
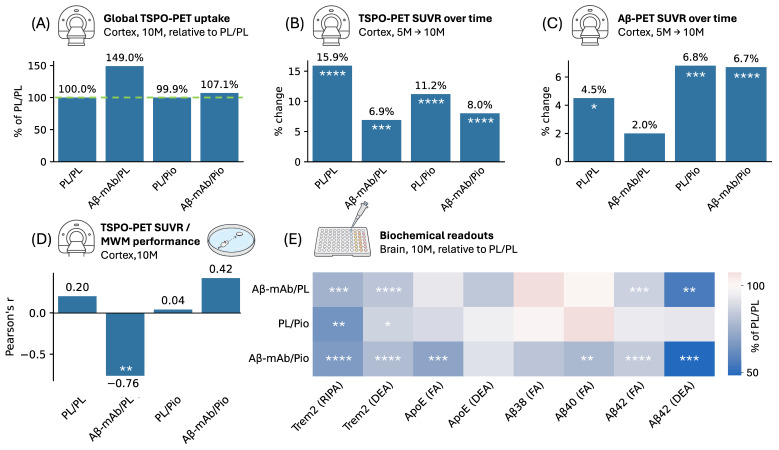
Integrated graphical summary of treatment-associated imaging, behavioral, and biochemical findings in App*^NL-G-F^* mice. (**A**) Mean global cortical TSPO-PET uptake at 10 months, expressed relative to the mean of the App*^NL-G-F^* double-placebo group (PL/PL, dashed green line = 100%). Anti-Aβ-mAb monotherapy showed the highest global cortical TSPO-PET signal (non-significant), whereas pioglitazone-containing treatment arms showed values closer to PL/PL. (**B**) Mean longitudinal percentage change in cortical TSPO-PET SUVR from 5 to 10 months. All active treatment arms showed a smaller increase than PL/PL. (**C**) Mean longitudinal percentage change in cortical Aβ-PET SUVR from 5 to 10 months. Pioglitazone-containing treatment arms showed increased Aβ-PET SUVR over time. (**D**) Pearson’s correlation coefficients between cortical TSPO-PET SUVR and Morris water maze performance at 10 months. A negative association was observed only in the anti-Aβ-mAb monotherapy arm. (**E**) Terminal biochemical readouts in brain tissue at 10 months, expressed relative to PL/PL. The heatmap summarizes treatment-associated changes (median value) in triggering receptor expressed on myeloid cells 2 (Trem2), apolipoprotein E (ApoE), and Aβ species across biochemical fractions. PL/PL, double placebo; Aβ-mAb/PL, anti-Aβ monoclonal antibody monotherapy; PL/Pio, pioglitazone monotherapy; Aβ-mAb/Pio, combination treatment with anti-Aβ-mAb and pioglitazone; MWM, Morris water maze; RIPA, radio-immuno-precipitation assay buffer; DEA, diethylamine buffer; FA, formic acid. Statistical significance: * *p* < 0.05, ** *p* < 0.01, *** *p* < 0.001, **** *p* < 0.0001.

Together, our data indicate that longitudinal TSPO-PET captures treatment-associated neuroinflammatory dynamics during anti-Aβ-mAb therapy in App*^NL-G-F^* mice, while pioglitazone co-treatment modifies this response in parallel with changes in Aβ-PET, biochemical markers, and behavioral associations, as summarized in Figure 5.

In an analysis of global brain TSPO signal, we observed a descriptive pattern compatible with a lower Aβ-mAb-associated TSPO-PET signal in the App*^NL-G-F^* mice receiving pioglitazone co-treatment; however, the respective treatment-arm contrasts did not remain significant after FDR correction and should therefore be interpreted only as exploratory. This is consistent with the possibility that pioglitazone may modulate the inflammatory side effects induced by immunotherapeutic Aβ-targeting strategies, but this requires confirmation in adequately powered studies. These initial global TSPO-PET observations are based on %ID, whereas the treatment-related longitudinal and regional analyses discussed below rely on SUVR-based quantification. In the more robust longitudinal TSPO-PET SUVR analysis, monotherapy with anti-Aβ-mAb or pioglitazone, as well as the combination treatment, delayed the increase of cortical TSPO-PET signal and reduced the magnitude of TSPO-associated neuroinflammatory changes in App*^NL-G-F^* mice. Longitudinal Aβ-PET SUVR analysis demonstrated no significant increase in Aβ-PET signal at either 7.5 or 10 months in the anti-Aβ monotherapy arm, consistent with antibody-mediated reduction of tracer-binding Aβ species [19]. By contrast, pioglitazone- and double-treated mice showed a robust increase in cortical [^18^F]FBB signal by 7.5 months, in line with previous observations of higher plaque compactness after treatment with PPARγ agonists [16]. However, this interpretation remains provisional. The in vivo Aβ-PET signal may reflect a composite of fibrillar and non-fibrillar plaque components, and ligand binding can vary with plaque molecular architecture and the availability of tracer-binding sites [20]. Moreover, [^18^F]FBB binds to both neuritic and diffuse fibrillar plaques [21]. The biochemical reduction of insoluble Aβ species and insoluble ApoE in the combination treatment group argues against a simple increase in total amyloid burden, particularly since ApoE promotes amyloid core deposition [22,23]. Another plausible contributor is redistribution of Aβ toward vascular deposits, since amyloid PET cannot distinguish parenchymal plaque Aβ from vascular Aβ in cerebral amyloid angiopathy (CAA) [24], and anti-Aβ immunotherapy has been associated in mouse models with increased vascular amyloid, microhemorrhages, vascular permeability, and altered cerebrovascular structure around vascular amyloid deposits [7,8,9]. Thus, the increased [^18^F]FBB signal in pioglitazone-treated mice should be interpreted cautiously as compatible with, but not specific for, plaque compaction/higher fibrillarity. Further studies will need histological validation, CAA and plaque-associated microglial readouts to distinguish between these possibilities.

Additionally, we found that mice with low cortical TSPO-PET SUVR at 5 months tended to have a higher percentage increase in microglial activation compared to those with high baseline TSPO-PET. Together with our previous work [17], these findings support the concept that baseline TSPO-PET signal may help stratify neuroinflammatory treatment responses in AD models. All active treatments reduced this baseline-change coupling, which suggests that treatment reduces the dependence of future TSPO-PET signal increases on baseline TSPO levels. However, this baseline-change analysis should be interpreted cautiously, since percentage change is mathematically related to the baseline value and may be influenced by regression to the mean. The weaker coupling might also reflect differences in variance, dynamic range, sample size, and/or missing scans across groups.

The possibility that pioglitazone modulates neuroinflammatory responses, even when administered in combination with Aβ-directed therapies, is supported by prior mechanistic studies showing that PPARγ activation can enhance Aβ uptake or degradation through CD36- and LXR/ApoE-dependent pathways in microglia and astrocytes [16,25]. By modulating inflammation, pioglitazone may facilitate cognitive improvement and possibly shift Aβ plaques toward a more fibrillar state that microglia can more readily handle; however, the present study does not directly define the underlying cellular phenotype.

Methodologically, our study reinforces the value of longitudinal TSPO-PET for tracing progressive neuroinflammatory changes. Coupling these imaging results with both biochemical and behavioral measures supports TSPO-PET as a useful tool for monitoring TSPO-associated neuroinflammatory changes in vivo. From a translational perspective, however, these findings should be considered hypothesis-generating rather than a direct rationale for immediate clinical combination trials. Although pioglitazone was generally well-tolerated in a small pilot study in nondiabetic patients with AD [26], larger clinical studies have not demonstrated clear efficacy; notably, the phase 3 TOMMORROW trial did not show that low-dose pioglitazone delayed the onset of mild cognitive impairment due to AD [27]. In addition, pioglitazone has known adverse-effect liabilities, including fluid retention and edema, increased incidence of heart failure, weight gain [28], and fracture risk [29], which are particularly relevant in older patients with AD and multimorbidity. Therefore, any future exploration of PPARγ agonist co-therapy would require careful patient selection, dose optimization, and prospective safety monitoring, rather than assuming broad clinical suitability. Translational extrapolation is further limited by the fact that the antibody used in this work differs from currently approved anti-Aβ antibodies. Lecanemab and donanemab target different Aβ species and have agent-specific dosing, titration, and adverse-event profiles, including distinct ARIA risk patterns [4,30,31]. More broadly, anti-Aβ antibody-associated risk is influenced by antibody characteristics, dose, and cerebrovascular amyloid burden [32,33]; thus, our results should be interpreted as supporting the general concept that immunomodulation may modify treatment-associated neuroinflammatory responses during anti-Aβ therapy, rather than as directly predicting the efficacy or safety of combining pioglitazone with currently used clinical antibodies in patients.

Importantly, our data show that simultaneous monitoring of Aβ-PET signal and TSPO-associated neuroinflammatory signal, combined with multi-regional analyses, is a powerful approach for understanding treatment-related changes in AD pathophysiology. This approach was also successfully translated into human imaging [34]. In the current work, we observed stronger regional associations between TSPO- and Aβ-PET signals during chronic administration of anti-Aβ-mAb or pioglitazone, which may be compatible with the ability of both treatment strategies to enhance the phagocytic capacity of microglia in response to amyloid pathology [35,36]. Interestingly, the dual-treatment approach showed the most sustained regional association between TSPO- and Aβ-PET signals at 10 months of age, which may suggest beneficial enhancement of microglial function by shifting microglia to an anti-inflammatory phenotype while priming them against Aβ plaques. However, in the absence of immunohistochemistry, TSPO colocalization, and plaque-associated microglial readouts, these results should be interpreted with caution.

Building on multiregional PET evaluation, the applied microglia desynchronization index (DI) based on TSPO-PET connectivity or, more broadly, the connectivity deviation score (CDS), may provide complementary information beyond conventional semiquantitative PET. Our previous work [18] provides the main biological rationale for this approach: near-complete pharmacological microglia depletion with PLX5622 substantially reduced TSPO-PET interregional correlations, altered synchronicity was observed across models of dysfunctional microglia and AD pathology, and single-cell radiotracing identified microglia as the dominant cellular contributor to the regionally desynchronized TSPO-PET signal. Nevertheless, DI remains a PET-derived covariance-based metric and should not be interpreted in this study as a direct standalone readout of microglial phenotype or function. Future studies combining DI with histology, cell-specific assays, and external replication will be needed to clarify its robustness and mechanistic interpretation further. CDS metrics derived from other tracers may provide additional clinical value, but they remain to be investigated and biologically interpreted. For instance, future studies of Aβ-PET-based CDS and its combination with the TSPO-PET DI may be promising for predicting clinical outcomes.

Finally, we attempted to link TSPO-PET endpoints with individual outcomes in behavioral testing. We observed a strong negative correlation between the magnitude of the TSPO-PET indices and time spent in the target quadrant, a proxy for cognitive performance in mice, in the anti-Aβ monotherapy group. However, this subgroup-specific association should be interpreted cautiously. The absence of comparable correlations in the pioglitazone-containing groups does not by itself demonstrate that pioglitazone prevented detrimental neuroinflammatory effects, since alternative explanations such as reduced variance, ceiling/floor effects, or narrower dynamic range in treated groups are also possible. Formal between-group comparison of regression slopes was not performed in the present study and will be important in future work.

Large parts of this work were conducted shortly before and during the COVID-19 pandemic, which led to the lack of immunohistochemistry and variable sample sizes across timepoints, especially at 7.5 months, mainly due to temporary interruptions in preclinical radiochemistry and radiosynthesis, condensed WT treatment arms, and a delay in publication. Thus, the used anti-Aβ-mAb is already outdated by successful phase III trials and market approval of the more specific anti-Aβ-mAbs lecanemab and donanemab, although we anticipate the general mechanism of anti-Aβ-therapy-induced neuroinflammation to be similar. In addition, the main focus of this work relates to neuroinflammation imaging during anti-Aβ-mAb treatment, which was successfully acquired and evaluated. Additional scans were excluded according to predefined technical criteria, as described in the Section 4. We used LMEMs with random intercepts to include all available observations without imputation; however, reduced sample sizes decreased the statistical power of the longitudinal analysis. Since most of the missing scans resulted from COVID-19-related interruptions rather than from animal dropout or treatment-related health status, major outcome-dependent missingness is unlikely, but cannot be fully excluded; thus, longitudinal effects should be interpreted with caution. A further limitation is that behavioral testing was performed only at the terminal time point. We opted for this design because cognitive deficits in App*^NL-G-F^* mice are mild and variably reported at earlier ages, with some studies finding no robust impairment at 6 months [37] and others detecting deficits from 6 months onward [38]. Thus, repeated longitudinal Morris water maze testing might have added burden while masking subtle effects through task familiarity.

Sex represents an additional potential source of heterogeneity in the present study, particularly for TSPO-PET trajectories and behavioral associations. Both female and male mice were included, but the study was not designed or powered for reliable sex-stratified treatment-effect analyses. This limitation is relevant because our previous longitudinal TSPO-PET study showed attenuation of TSPO-PET signal progression in pioglitazone-treated females, but an opposite tendency in treated males [17]. Human AD data likewise suggest sex-related differences in TSPO-PET signal, particularly more pronounced Aβ-plaque-independent neuroinflammatory responses in women, which may be related to increased tau accumulation [34]. Future studies should be prospectively powered to test sex-treatment-time interactions.

The absence of histological and functional follow-up is particularly relevant for interpretation of TSPO-PET, as the signal is not fully microglia-specific and may also include astrocytic or vascular contributions [10]. Moreover, TSPO-PET does not distinguish microglial phenotypes or directly measure phagocytic activity. Accordingly, the present findings should be interpreted as treatment-associated changes in neuroinflammatory signal rather than direct evidence of beneficial or harmful microglial function. The interpretation of increased [^18^F]FBB signal under pioglitazone as plaque compaction or higher fibrillarity remains provisional.

## 4. Materials and Methods

### 4.1. Experimental Design

This study included a total of 72 C57BL/6 mice with two genotypes: App*^NL-G-F^* (APP knock-in [39], *n* = 56) and wild-type (WT, *n* = 16). The sample size was chosen based on prior experience. WT mice were split into two treatment arms: double placebo (PL/PL, *n* = 8) and anti-Aβ antibodies (Aβ-mAb/PL, *n* = 8). App*^NL-G-F^* mice had the same treatment arms (PL/PL, *n* = 10; Aβ-mAb/PL, *n* = 14) and two additional arms: pioglitazone (PL/Pio, *n* = 11) and the combination of anti-Aβ antibodies and pioglitazone (Aβ-mAb/Pio, *n* = 21) (Table S7). The mice were then scanned with TSPO- and Aβ-PET at 5, 7.5, and 10 months of age. We selected 5 months of age as the baseline because this stage precedes advanced pathology while already providing a quantifiable in vivo PET signal [12]. To assess Aβ-mAb-induced neuroinflammation in App*^NL-G-F^* mice and answer the question of whether and how pioglitazone modulates it, we analyzed longitudinal TSPO- and Aβ-PET uptake in the cortex. To assess the effect of different treatment options on regional Aβ-PET signal in the mouse brain, we correlated TSPO-PET with Aβ-PET uptake in 20 predefined atlas brain regions and estimated the slope of a linear fit. Finally, we compared behavioral parameters between the treatment arms and correlated these values with TSPO-PET uptake and microglia DI, a personalized parameter reflecting the deviation of individual TSPO uptake patterns from the TSPO-PET connectivity of the control cohort [18].

#### 4.1.1. Randomization

Randomization procedures were implemented throughout the study. Animals were randomly assigned to the treatment groups. Mice assigned to pioglitazone treatment were distributed across multiple cages to enable administration via pioglitazone-containing chow while minimizing potential cage effects. Animals were further randomly scheduled for imaging sessions, randomly positioned in the animal bed (4 mice per scan) during scanning, and randomly scheduled for behavioral assessments.

#### 4.1.2. Blinding

Complete blinding of the primary experimenter could not be maintained in this study, as one experimenter was responsible for treatment administration, imaging, behavioral testing, and initial data processing and was therefore aware of group allocation. To reduce potential bias, randomization procedures were applied throughout the study, and confirmation of data quality and outcome interpretation was performed by an additional blinded investigator and an independent automated analysis pipeline. Manual preprocessing of imaging data was likewise verified by a blinded reviewer and an automated approach.

### 4.2. Animals

This manuscript is reported in accordance with the ARRIVE 2.0 Essential 10 guidelines for the reporting of animal research. All animal experiments were approved by the local animal care committee of the Government of Upper Bavaria (Regierung von Oberbayern, approval numbers ROB-55.2-2532.Vet_02-15-210 and ROB-55.2-2532.Vet_02-19-26, approval date 05.09.2019) and conducted in accordance with applicable national and international regulations, including the German Animal Welfare Act, the U.K. Animals (Scientific Procedures) Act 1986, and EU Directive 2010/63/EU.

Female and male App*^NL-G-F^* mice and C57BL/6 control (WT) mice were used. Animals were group-housed under specific-pathogen-free conditions in a temperature- and humidity-controlled environment with a 12 h/12 h light-dark cycle, with ad libitum access to water and standard chow (Ssniff Ms-H, Ssniff Spezialdiäten GmbH, Soest, Germany). Each individual mouse constituted one experimental unit. All experimental procedures were performed at the Department of Nuclear Medicine, LMU University Hospital (LMU Munich, Munich, Germany).

App*^NL-G-F^* mice were generated on a C57BL/6 background, carrying three humanized mutations associated with familial AD (Swedish KM670/671NL, Beyreuther/Iberian I716F, and Arctic G mutations). This model closely recapitulates human amyloid pathology, with Aβ deposition detectable from approximately 2 months of age by histological assessment [39] and from around 5 months onward by Aβ-PET [12]. Measurable memory impairments in this model have been reported from approximately 6 months of age, although their onset and magnitude vary across studies [37,38].

### 4.3. Treatment

At baseline (5 months of age), WT mice were treated with β1 mouse monoclonal IgG2a antibody (Aβ-mAb) [40] (WT Aβ-mAb/PL cohort) or placebo (PL) (WT PL/PL cohort). In App*^NL-G-F^* mice, the same two groups were studied, as well as two additional treatment options: pioglitazone (Pio, App*^NL-G-F^* PL/Pio cohort) and combination treatment with Aβ-mAb and Pio (App*^NL-G-F^* Aβ-mAb/Pio cohort).

For antibody treatment, the Aβ-mAb was prepared in 90 mM NaCl and 50 mM Tris, pH 7.1, yielding a solution concentration of 2 mg/mL. Each mouse in the Aβ-mAb and Aβ-mAb/Pio cohorts received a weekly intraperitoneal injection of 0.5 mg Aβ-mAb, with an injection volume of approximately 10 mL/kg body weight. The Aβ-mAb was generously provided by Novartis Pharma AG, Basel, Switzerland. The β1 mouse monoclonal IgG2a recognizes amino acids 3–6 of human Aβ and binds a defined linear sequence on the N-terminus of the human Aβ peptide, specifically the tetrapeptide EFRH of human Aβ. Importantly, the Aβ-mAb does not bind the corresponding region in mouse Aβ, where the sequence differs (mouse Aβ shows EFGH in that position), which makes it specific to human Aβ in transgenic mouse models. Control animals received corresponding placebo intraperitoneal injections with NaCl solution.

Pioglitazone was incorporated into the regular mouse chow and administered daily via the mice’s ad libitum diet. Mice assigned to pioglitazone treatment were housed accordingly, distributed across multiple cages, and food intake was monitored by weekly weighing of the pioglitazone-containing chow to ensure comparable consumption across cages. Both the Aβ-mAb and pioglitazone treatments were initiated following the completion of baseline TSPO- and Aβ-PET scans and continued until all study procedures were completed. Standard mouse chow served as a dietary placebo for the control groups.

### 4.4. Behavioral Testing

Prior to the final scanning time point, at 10 months of age, each cohort underwent behavioral assessment using an MWM to avoid potential effects of anesthesia on behavioral testing. MWM was conducted according to a standard protocol, slightly modified based on spatial conditions and prior experience. In brief, a circular pool was filled with water and contained a platform submerged just below the water surface in one quadrant so that the platform was not immediately visible to the mice. Patterns on the walls of the pool were used as visual cues to help the mice orient themselves and find the platform. The procedure of the behavioral testing was divided into three parts. During the habituation phase (day 0), the mice were first introduced to the pool containing a visible platform, which could be easily found to reduce stress and allow familiarization with the environment. During the training phase (days 1 to 5), the mice were placed into opaque water made cloudy with non-toxic paint, so that they could learn to locate the hidden platform using spatial cues. The mice were placed into the pool from different starting points. The third part of the procedure, the probe trial (day 6), included removal of the platform to track searching by individual mice. The time spent in the target quadrant where the platform used to be was tracked as an index of memory retention. To maintain consistent and tolerable conditions for each mouse, the water temperature was checked frequently and kept at 24 °C at all times.

### 4.5. Analysis of AD Signature Proteins

After completing the second follow-up scan (FU2), at 10 months of age, mice were transcardially perfused with phosphate-buffered saline (PBS) to harvest the brains. The brain was flash-frozen in liquid nitrogen for subsequent biochemical analyses.

To prepare the fractions for biochemical analyses, the PBS-perfused and flash-frozen brains were kept in a −80 °C freezer until further processing. The brain homogenates for TREM2, Aβ, and ApoE quantification were essentially prepared as described previously [41]. DEA (0.2% Diethylamine in 50 mM NaCl, pH 10) and RIPA lysates (20 mM Tris-HCl pH 7.5, 150 mM NaCl, 1 mM Na2EDTA, 1% NP-40, 1% sodium deoxycholate, 2.5 mM sodium pyrophosphate) were prepared from brain hemispheres by ultracentrifugation. The RIPA insoluble material of one hemisphere was homogenized in 400 µL 70% formic acid (FA fraction). The FA fraction was neutralized with 20 × 1 M Tris-HCl buffer at pH 9.5 and used for ApoE and Aβ analysis. The protein extraction protocol schematic is displayed in Figure S5.

ELISA quantification for Aβ and ApoE was performed as described previously [41]. In brief, Aβ contained in DEA and FA fractions was quantified by a sandwich immunoassay using the MESO Scale Aβ Triplex plates and Discovery SECTOR Imager 2400 (Meso Scale Diagnostics, LLC, Rockville, MD, USA) as described previously [42]. Samples were measured in triplicate. ApoE contained in DEA and FA fractions was quantified by a sandwich immunoassay using the Mesoscale Streptavidin plates and Discovery.

SECTOR Imager 2400 with adjusted dilutions using the R-Plex ApoE antibody set F212L (Meso Scale Diagnostics, LLC, Rockville, MD, USA). The samples were measured in three technical replicates.

### 4.6. PET Imaging and Analysis

All small-animal PET (μPET) procedures followed an established standardized protocol for radiochemistry, acquisition, and post-processing [43,44]. PET imaging was performed on a Siemens Inveon DPET (Siemens Healthineers, Erlangen, Germany), followed by a 7 min transmission scan using a rotating [^57^Co] point source. Briefly, we used [^18^F]GE-180 TSPO μPET with an emission window of 60–90 min post-injection to measure cerebral microglial activity and [^18^F]FBB Aβ-μPET with an emission window of 30–60 min post-injection to quantify the amount of fibrillar amyloid in the mouse brain. Table S7 provides a detailed description of the number of scans performed in each cohort. All μPET experiments were performed with isoflurane anesthesia (1.5% at time of tracer injection and during imaging; delivery 3.5 L/min). The mice were scanned at 5 (baseline), 7.5, and 10 months of age (both TSPO- and Aβ-μPET, except for WT mice at 5 and 7.5 months, which only received TSPO-PET), resulting in a total of 165 TSPO-PET and 154 Aβ-PET scans. PET data were visually inspected and spatially registered to respective in-house tracer-specific templates [45] using a user-independent automated elastic transformation in PMOD software (Fusion tool, version 3.5, PMOD Technologies, Zurich, Switzerland) to exclude operator bias.

For [^18^F]GE-180 TSPO-PET, we used different normalization approaches depending on the analysis aim. For global comparisons between genotypes and treatment arms, we quantified TSPO uptake as %ID to avoid potential bias from changes in the pseudo-reference region. For longitudinal and regional analyses, including connectivity and desynchronization metrics, we expressed TSPO uptake as SUVR. A data-driven approach was applied to [^18^F]GE-180 data to find the best pseudo-reference region for SUVR calculation: the distributions of [^18^F]GE-180 uptake (%ID) were compared in every volume of interest (VOI) of both pooled WT cohorts and pooled App*^NL-G-F^* cohorts, and the VOI with the smallest effect size (Cohen’s d) was selected. Based on this screening procedure, the brain stem showed the smallest genotype-related difference and was therefore used as a pseudo-reference region (Table S1, Figure S3). Additionally, we generated z-score images using 10-month TSPO-PET SUVR images from *n* = 14 WT mice treated with PL/PL or Aβ-mAb/PL as the reference. For the [^18^F]FBB data, we consistently used SUVR with periaqueductal gray matter as the pseudo-reference region [12], and Aβ z-score images were computed from these SUVR maps. Image parcellation was performed using Ma-Benveniste-Mirrione mouse brain atlas [46], with further subdivision of the neocortical target region (visual, auditory, entorhinal, sensorimotor, and somatosensory cortex, all split into left and right). Table S6 provides further description of the VOIs used in the study. Cerebellum, brain stem, and right and left sensorimotor cortex VOIs were manually cropped to correct for signal spill-in from the brain ventricles and Harderian glands. All processing steps except spatial registration were performed using a custom Python script (version 3.12, NiBabel package [47]).

During the COVID-19 pandemic, temporary lockdown-related restrictions and interruptions in preclinical radiochemistry and radiosynthesis limited the acquisition of some planned PET scans, particularly at the 7.5-month timepoint. Thus, not all missing data points reflect failed imaging procedures; in many cases, the scans could not be performed as scheduled. For acquired scans, exclusion criteria were predefined and included insufficient tracer uptake (<10 MBq), paravenous tracer administration, and technical malfunction of the imaging system. When these criteria were met, only the affected scans were excluded from the analysis, while the animals remained in the study. Consequently, the number of animals contributing data varied between timepoints.

### 4.7. Individual Assessment of Microglia Desynchronization

Using the SUVR images, interregional correlation coefficients (ICCs) were calculated for mice at 10 months of age using a molecular connectivity Python package developed in our department [48], following the previously validated TSPO-PET-based microglia desynchronization framework described in [18]: briefly, Pearson’s correlation coefficient between all VOI pairs was computed, followed by the Fisher’s R to Z transformation [49] and bootstrapping by resampling with replacement. Individual connectivity deviation scores (CDS, called desynchronization index [DI] for [^18^F]GE-180 [18]) were calculated for each VOI*_i_* by: (1) estimating bootstrapped linear fit for every VOI pair of the control cohort (pooled WT PL/PL and WT Aβ-mAb/PL cohort to increase robustness, *n* = 14); (2) for each VOI pair of each study subject, calculating the perpendicular distance from the subject’s pair of values to the corresponding linear fit; and (3) summing up the perpendicular distances of all the pairs that included the VOI*_i_*. To obtain the DIs of composite VOIs, such as whole cortex and EHA, PC1 was calculated on the DIs of the constituent VOIs (Table S6), as described in [18] (DI PC1).

### 4.8. Statistical Analysis

The Shapiro-Wilk normality test [50] was performed on all distributions before comparisons. To test for differences in TSPO-PET (%ID), Kruskal-Wallis H-tests and Mann-Whitney U (MWU) tests [51] (two-sided, adjusted by FDR [52]) were performed using the Pingouin statistical library (Python 3.12). To test for differences in time spent in the target quadrant and the levels of TREM2 (RIPA), Aβ38 (FA), and Aβ42 (DEA) at 10 months, MWU tests (two-sided, adjusted by FDR) were performed using statsannotations statistical library (Python 3.12). To test for differences in the levels of TREM2 (DEA), ApoE, Aβ40 (FA), and Aβ42 (FA), unpaired t-tests (two-tailed, adjusted by FDR) were performed using the statsannotations statistical library (Python 3.12).

Since the dataset was unbalanced across groups and time points, longitudinal analyses were performed using LMEMs with random intercepts for individual mice, allowing inclusion of all available observations without imputation. To compare TSPO- and Aβ-PET SUVR across genotype, time point, and treatment type, we applied LMEMs, type III ANOVA with Satterthwaite’s method, and post-hoc contrasts based on estimated marginal means, all of which were implemented in R (version 4.4.1, lme4 and emmeans libraries).

Sample sizes for each endpoint, group, and time point are reported in the corresponding figure legends and in Table S7. Post-hoc pairwise contrasts were adjusted for multiple comparisons using FDR correction within the respective analysis. In all statistical tests, *p* < 0.05 was used as the significance threshold. Findings with FDR-adjusted *p* ≥ 0.05 were interpreted as descriptive or exploratory and were not used as confirmatory evidence of treatment effects.

## 5. Conclusions

In conclusion, our study highlights the potential of TSPO-PET to monitor neuroinflammation during Aβ-related treatments in AD. Dual treatment strategies combining Aβ-targeting antibodies and immunomodulators may influence treatment-associated neuroinflammatory responses and potentially improve side effect profiles of anti-Aβ therapy, thereby warranting further evaluation with cell-specific and functional validation.

## Data Availability

The original contributions presented in this study are included in the article/Supplementary Material. Further inquiries can be directed to the corresponding author.

## Supplementary Materials

The following supporting information can be downloaded at https://www.mdpi.com/article/10.3390/ijms27104632/s1.

## Abbreviations

The following abbreviations are used in this manuscript:

Aβ	β-amyloid
AD	Alzheimer’s disease
ANOVA	Analysis of variance
ApoE	Apolipoprotein E
ARIA	Amyloid-related imaging abnormalities
CAA	Cerebral amyloid angiopathy
CDS	Connectivity deviation score
DEA	Diethylamine buffer
DI	Desynchronization index
EHA	Entorhinal-hippocampus-amygdala region
ELISA	Enzyme-linked immunosorbent assay
FA	Formic acid
FBB	Florbetaben
FDR	False discovery rate
ICC	Interregional correlation coefficient
IQR	Interquartile range
LMEM	Linear mixed-effects model
mAb	Monoclonal antibody
MWM	Morris water maze
MWU	Mann-Whitney U-test
PBS	Phosphate-buffered saline
PC1	First principal component
PET	Positron emission tomography
Pio	Pioglitazone
PL	Placebo
PPARγ	Peroxisome proliferator-activated receptor γ
RIPA	Radio-immuno-precipitation assay buffer
SUVR	Standardized uptake value ratio
TREM2	Triggering receptor expressed on myeloid cells 2
TSPO	18 kDa translocator protein
VOI	Volume of interest
WT	Wild type
μPET	Small-animal positron emission tomography
%ID	Percentage of injected dose per cubic centimeter
