# Supplementary material for: Tracking of Neuroinflammation Dynamics During Combined Anti-β-Amyloid Therapy (AAT) and Immunomodulation in a Preclinical Alzheimer’s Disease Model
Source: Int J Mol Sci. 2026 May 21;27(10):4632. doi: 10.3390/ijms27104632 (PMC13206848; doi:10.3390/ijms27104632)
Supplement: Supplementary file 1 [file ijms-27-04632-s001.zip › ijms-4270293-supplementary.pdf]

Supplementary Materials for

# Tracking of Neuroinflammation Dynamics During Combined Anti-β-Amyloid Therapy (AAT) and Immunomodulation in a Preclinical Alzheimer's Disease Model

Karin Wind-Mark et al.

*Corresponding author: Matthias Brendel, E-mail: Matthias.Brendel@med.uni-muenchen.de

**This PDF file includes:**

Supplementary Figs. S1 to S5

Supplementary Tables S1 to S7

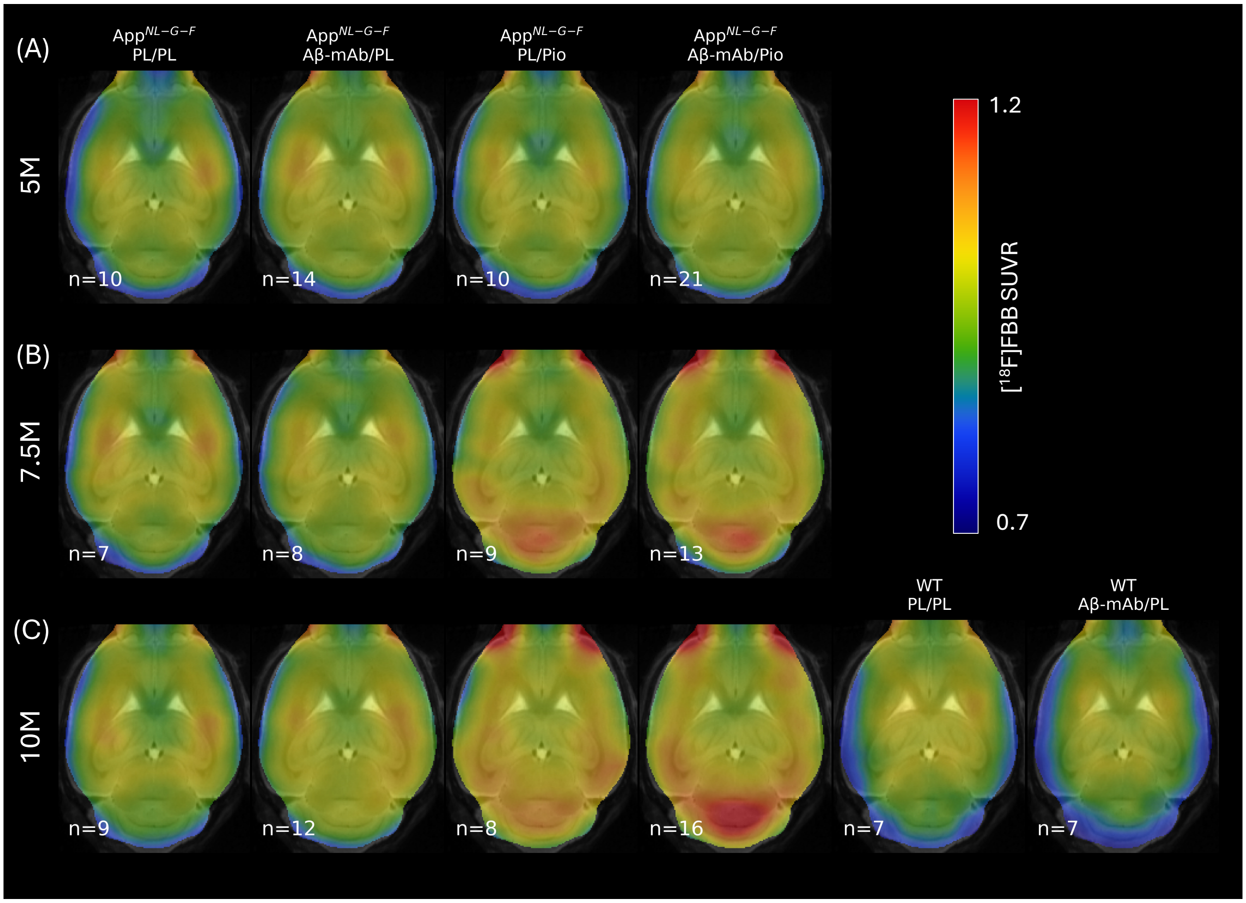


***Figure S1***. *Voxelwise group average Aβ-PET images (scaled by mean uptake in periaqueductal gray matter) generated from all animals within each cohort at (A) 5 months, (B) 7.5 months, and (C) 10 months of age. Treatment arms: PL/PL – double placebo, Aβ-mAb/PL – anti-β-amyloid antibodies, PL/Pio – pioglitazone, Aβ-mAb/Pio – combination of anti-β-amyloid antibodies and pioglitazone. WT – wild-type mice, App$^{NL\text{-}G\text{-}F}$ – APP knock-in mice. The number of mice in respective cohorts (n) is shown in the bottom left corner.*

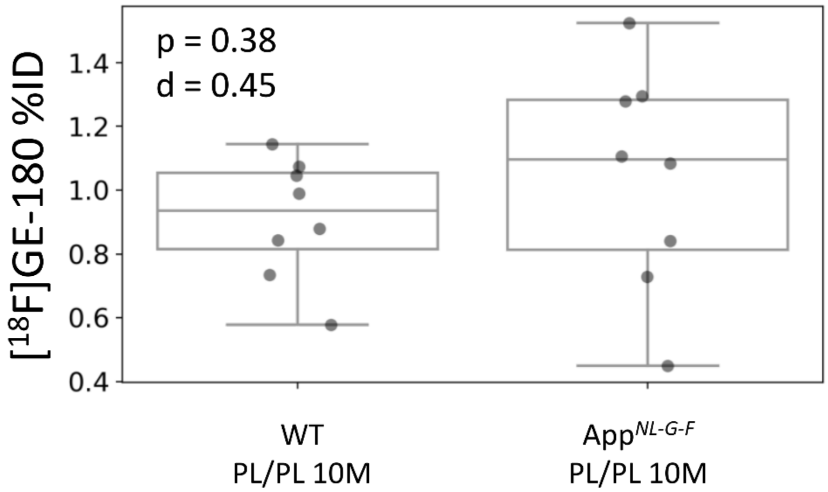


***Figure S2***. *[$^{18}$F]GE-180 %ID in brain stem of App$^{NL\text{-}G\text{-}F}$ and WT mice treated with PL/PL at 10 months of age. Boxes show the IQR and median; whiskers follow the 1.5×IQR rule.*

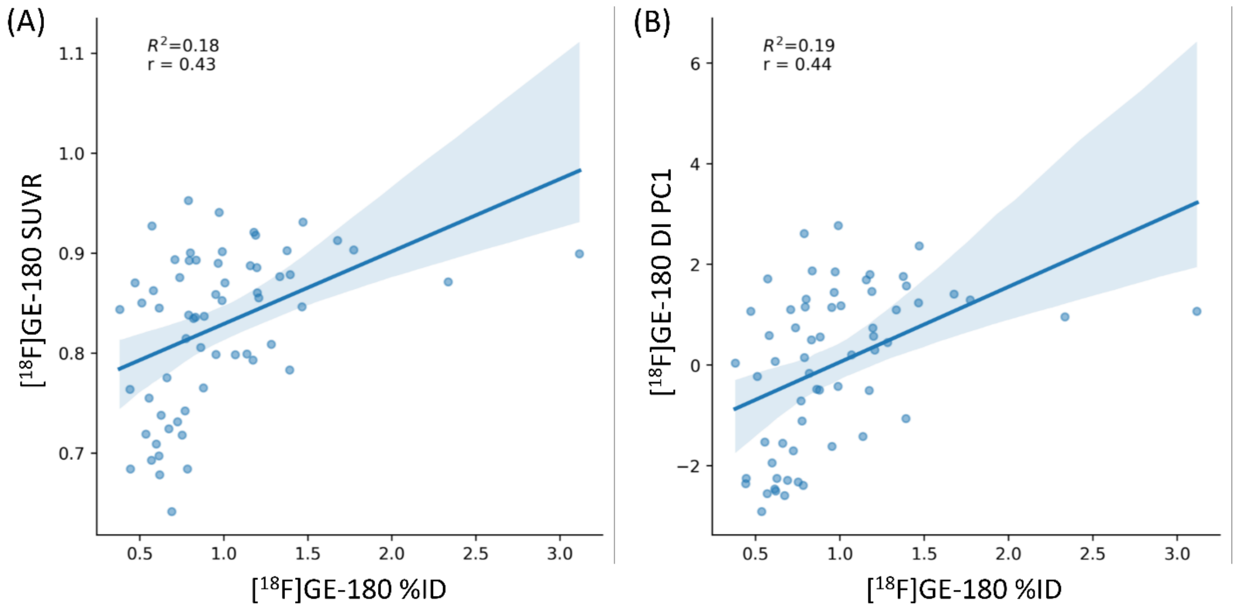


***Figure S3.*** *Correlation between %ID-normalized mean cortical uptake in investigated mice (10 months of age) and corresponding (**A**) mean SUVR (scaled to brain stem uptake), (**B**) SUVR-based DI (PC1).*

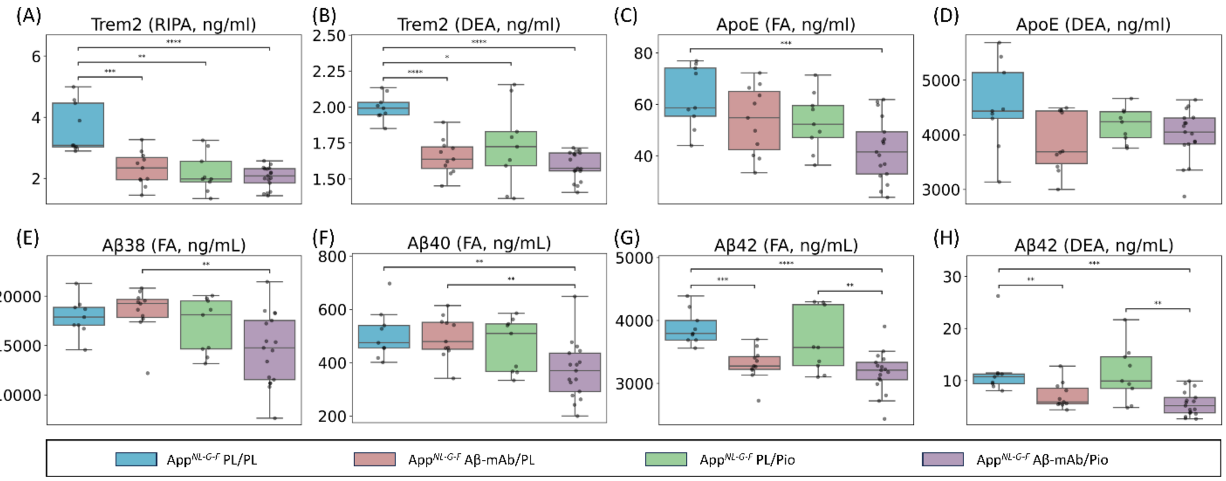


***Figure S4.*** *Biochemical analyses at 10 months of age.* ***(A, B)*** *Trem2 levels in App$^{NL\text{-}G\text{-}F}$.* ***(C, D)*** *ApoE levels in App$^{NL\text{-}G\text{-}F}$.* ***(E-H)*** *A$\beta$ levels in App$^{NL\text{-}G\text{-}F}$. Statistical significance: *p < 0.05, **p < 0.01, ***p < 0.001, ****p < 0.0001. Boxes show the IQR and median; whiskers follow the 1.5×IQR rule.*

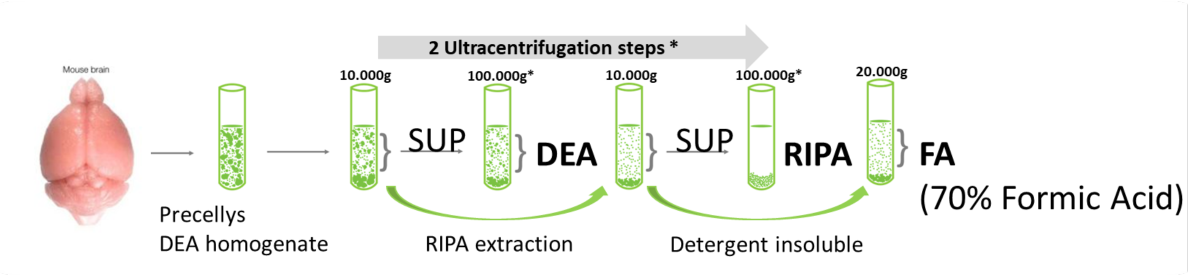


***Figure S5.*** *Schematic view of the protein extraction protocol used to collect soluble brain proteins (DEA), detergent solubilized homogenates (RIPA) and formic acid extracted aggregates (FA) from mouse brain hemispheres.*

| Region | Ratio of cohort means | Cohen's d |
|---|---|---|
| Brain stem | 1.14 | 0.45 |
| Thalamus right | 1.15 | 0.48 |
| Thalamus left | 1.17 | 0.51 |
| Basal forebrain septum | 1.23 | 0.66 |
| Cerebellum | 1.25 | 0.79 |
| Striatum right | 1.28 | 0.80 |
| Striatum left | 1.30 | 0.86 |
| Hippocampus right | 1.31 | 0.86 |
| Amygdala right | 1.35 | 0.90 |
| Amygdala left | 1.35 | 0.98 |
| Entorhinal cortex right | 1.36 | 0.94 |
| Hippocampus left | 1.36 | 1.00 |
| Entorhinal cortex left | 1.38 | 1.12 |
| Somatomotor cortex right | 1.40 | 1.00 |
| Auditory cortex left | 1.44 | 1.15 |
| Auditory cortex right | 1.45 | 1.09 |
| Somatomotor cortex left | 1.46 | 1.13 |
| Visual cortex right | 1.51 | 1.19 |
| Somatosensory cortex left | 1.54 | 1.27 |
| Visual cortex left | 1.59 | 1.31 |
| Somatosensory cortex right | 1.60 | 1.31 |

***Table S1**. Ratio of [$^{18}$F]GE-180 %ID mean values in each investigated brain region and Cohen's d effect size comparing App$^{NL\text{-}G\text{-}F}$ and WT mice treated with PL/PL at 10 months of age. Regions are sorted by the ratio of cohort means.*

| Effect | NumDF | DenDF | F value | Pr(>F) |
|---|---|---|---|---|
| Genotype | 1 | 59.814 | 115.644 | 1.32E-15 |
| Treatment | 3 | 59.759 | 0.331 | 0.80 |
| Timepoint | 2 | 89.259 | 19.002 | 1.33E-07 |
| Genotype:Treatment | 1 | 59.814 | 1.838 | 0.18 |
| Genotype:Timepoint | 2 | 90.591 | 16.791 | 6.27E-07 |
| Treatment:Timepoint | 6 | 90.836 | 1.311 | 0.26 |
| Genotype:Treatment:Timepoint | 2 | 90.591 | 1.939 | 0.15 |

***Table S2***. *Type III ANOVA results for the longitudinal TSPO-PET SUVR in cortex.*

| Genotype | Treatment | Timepoint | EMM (± SE) | 95% CI |
| --- | --- | --- | --- | --- |
| WT | PL/PL | 5M | 0.718 ± 0.015 | [0.689, 0.748] |
| $App^{NL-G-F}$ | PL/PL | 5M | 0.758 ± 0.013 | [0.733, 0.783] |
| WT | Ab-mAb/PL | 5M | 0.714 ± 0.014 | [0.686, 0.742] |
| $App^{NL-G-F}$ | Ab-mAb/PL | 5M | 0.807 ± 0.013 | [0.781, 0.833] |
| $App^{NL-G-F}$ | PL/Pio | 5M | 0.770 ± 0.012 | [0.747, 0.794] |
| $App^{NL-G-F}$ | Ab-mAb/Pio | 5M | 0.788 ± 0.010 | [0.770, 0.807] |
| WT | PL/PL | 7.5M | 0.725 ± 0.019 | [0.687, 0.763] |
| $App^{NL-G-F}$ | PL/PL | 7.5M | 0.823 ± 0.017 | [0.789, 0.857] |
| WT | Ab-mAb/PL | 7.5M | 0.696 ± 0.015 | [0.667, 0.726] |
| $App^{NL-G-F}$ | Ab-mAb/PL | 7.5M | 0.837 ± 0.017 | [0.803, 0.870] |
| $App^{NL-G-F}$ | PL/Pio | 7.5M | 0.823 ± 0.016 | [0.792, 0.854] |
| $App^{NL-G-F}$ | Ab-mAb/Pio | 7.5M | 0.819 ± 0.011 | [0.798, 0.840] |
| WT | PL/PL | 10M | 0.713 ± 0.014 | [0.685, 0.741] |
| $App^{NL-G-F}$ | PL/PL | 10M | 0.878 ± 0.014 | [0.850, 0.906] |
| WT | Ab-mAb/PL | 10M | 0.706 ± 0.016 | [0.674, 0.738] |
| $App^{NL-G-F}$ | Ab-mAb/PL | 10M | 0.863 ± 0.012 | [0.840, 0.885] |
| $App^{NL-G-F}$ | PL/Pio | 10M | 0.857 ± 0.013 | [0.832, 0.882] |
| $App^{NL-G-F}$ | Ab-mAb/Pio | 10M | 0.852 ± 0.009 | [0.833, 0.870] |

***Table S3***. *Estimated marginal means (EMMs) for the longitudinal TSPO-PET SUVR in cortex by genotype, treatment, and timepoint.*

| Effect | NumDF | DenDF | F value | Pr(>F) |
|---|---|---|---|---|
| Genotype | 1 | 132.510 | 12.6735 | 0.00052 |
| Treatment | 3 | 68.577 | 11.6628 | 2.90E-06 |
| Timepoint | 2 | 91.480 | 32.3175 | 2.42E-11 |
| Genotype:Treatment | 1 | 132.510 | 6.0399 | 0.015 |
| Treatment:Timepoint | 6 | 91.467 | 4.4240 | 0.00057 |

***Table S4***. *Type III ANOVA results for the longitudinal Aβ-PET SUVR in cortex.*

| Genotype | Treatment | Timepoint | EMM (± SE) | 95% CI |
|---|---|---|---|---|
| $App^{NL-G-F}$ | PL/PL | 5M | 0.874 ± 0.010 | [0.855, 0.894] |
| $App^{NL-G-F}$ | Ab-mAb/PL | 5M | 0.914 ± 0.008 | [0.897, 0.930] |
| $App^{NL-G-F}$ | PL/Pio | 5M | 0.895 ± 0.010 | [0.876, 0.914] |
| $App^{NL-G-F}$ | Ab-mAb/Pio | 5M | 0.904 ± 0.007 | [0.891, 0.918] |
| $App^{NL-G-F}$ | PL/PL | 7.5M | 0.913 ± 0.012 | [0.890, 0.936] |
| $App^{NL-G-F}$ | Ab-mAb/PL | 7.5M | 0.905 ± 0.011 | [0.884, 0.927] |
| $App^{NL-G-F}$ | PL/Pio | 7.5M | 0.956 ± 0.010 | [0.935, 0.976] |
| $App^{NL-G-F}$ | Ab-mAb/Pio | 7.5M | 0.966 ± 0.009 | [0.949, 0.983] |
| WT | PL/PL | 10M | 0.897 ± 0.012 | [0.874, 0.921] |
| $App^{NL-G-F}$ | PL/PL | 10M | 0.909 ± 0.010 | [0.889, 0.930] |
| WT | Ab-mAb/PL | 10M | 0.867 ± 0.012 | [0.844, 0.891] |
| $App^{NL-G-F}$ | Ab-mAb/PL | 10M | 0.932 ± 0.009 | [0.914, 0.950] |
| $App^{NL-G-F}$ | PL/Pio | 10M | 0.948 ± 0.011 | [0.926, 0.969] |
| $App^{NL-G-F}$ | Ab-mAb/Pio | 10M | 0.974 ± 0.008 | [0.959, 0.989] |

***Table S5***. *Estimated marginal means (EMMs) for the longitudinal Aβ-PET SUVR in cortex by genotype, treatment, and timepoint.*

| | List of VOIs |
|---|---|
| Cortex (DI PC1 calculation) | Sensorimotor Cortex (R, L), Somatosensory Cortex (R, L), Visual Cortex (R, L), Auditory Cortex (R, L) |
| Amygdala | Amygdala (R, L) |
| EHA | Entorhinal Cortex (R, L), Hippocampus (R, L), Amygdala (R, L) |
| VOIs for TSPO-Aβ z-score correlation calculation (Fig. 3) | Sensorimotor Cortex (R, L), Somatosensory Cortex (R, L), Visual Cortex (R, L), Auditory Cortex (R, L), Entorhinal Cortex (R, L), Hippocampus (R, L), Amygdala (R, L), Thalamus (R, L), Striatum (R, L), Cerebellum, Basal Forebrain Septum |

***Table S6.*** *Description of VOIs used in the study. R – right, L – left, DI – desynchronization index, PC1 – first principal component, EHA – entorhinal-hippocampus-amygdala region.*

| Genotype and treatment arm | Number of mice | Number of scans at 5M | | | Number of scans at 7.5M | | | Number of scans at 10M | | |
|---|---|---|---|---|---|---|---|---|---|---|
| | | [$^{18}$F]GE-180 | [$^{18}$F]FBB | Both scans | [$^{18}$F]GE-180 | [$^{18}$F]FBB | Both scans | [$^{18}$F]GE-180 | [$^{18}$F]FBB | Both scans |
| WT PL/PL | 8 | 7 | 0 | 0 | 4 | 0 | 0 | 8 | 7 | 7 |
| WT Aβ-mAb/PL | 8 | 8 | 0 | 0 | 7 | 0 | 0 | 6 | 7 | 5 |
| $App^{NL\text{-}G\text{-}F}$ PL/PL | 10 | 10 | 10 | 10 | 5 | 7 | 3 | 8 | 9 | 8 |
| $App^{NL\text{-}G\text{-}F}$ Aβ-mAb/PL | 14 | 9 | 14 | 9 | 5 | 8 | 2 | 12 | 12 | 11 |
| $App^{NL\text{-}G\text{-}F}$ PL/Pio | 11 | 11 | 10 | 10 | 6 | 9 | 6 | 10 | 8 | 8 |
| $App^{NL\text{-}G\text{-}F}$ Aβ-mAb/Pio | 21 | 17 | 21 | 17 | 13 | 13 | 7 | 18 | 16 | 15 |
| **Sum** | **72** | **62** | **55** | **46** | **40** | **37** | **18** | **62** | **59** | **54** |

***Table S7.*** *Baseline cohort allocation and number of mice contributing analyzable PET data at each timepoint. Treatment arms: PL/PL – double placebo, Aβ-mAb/PL – anti-β-amyloid antibodies, PL/Pio – pioglitazone, Aβ-mAb/Pio – combination of anti-β-amyloid antibodies and pioglitazone. WT – wild-type mice,* $App^{NL\text{-}G\text{-}F}$ *– APP knock-in mice.*
